# Licorice zinc suppresses melanogenesis via inhibiting the activation of P38MAPK and JNK signaling pathway in C57BL/6J mice skin

**DOI:** 10.1590/acb371002

**Published:** 2022-12-19

**Authors:** Jing-yan Wang, Xing-yu Xie, Ying Deng, Hong-qiu Yang, Xiao-shuang Du, Ping Liu, Yu Du

**Affiliations:** 1 Master. Southwest Medical University – Luzhou, Sichuan Province, People’s Republic of China; 2 Master. Southwest Medical University - Affiliated Traditional Chinese Medicine Hospital - Medical Cosmetic Center – Luzhou, Sichuan Province, People’s Republic of China.; 3 Bachelor. Medical University - Affiliated Traditional Chinese Medicine Hospital - Medical Cosmetic Center – Luzhou, Sichuan Province, People’s Republic of China.

**Keywords:** Melanosis, Glycyrrhiza, Zinc, MAP Kinase Signaling System

## Abstract

**Purpose:**

The active melanocytes in the skin were affected by hormones and ultraviolet (UV) irradiation. Licorice zinc has a whitening effect, which may have a prominent potential in the treatment of pigmented skin disease.

**Methods:**

Modeling chloasma C57BL/6J mice by daily progesterone injection (15 mg/kg) and ultraviolet B (UVB) irradiation (λ = 312 nm, 2 h/day) for 30 days. Then, mice were given 0.65, 1.3, and 2.6 (g/kg) of licorice zinc and tranexamic acid 250 mg daily by oral administration for 14 days, respectively. Hematoxylin and eosin and Fontana-Masson staining, and Western blotting (WB) were performed to test the inhibitory of melanogenesis and activation of c-Jun-N-terminal (JNK)/p38 mitogen-activated protein kinases (MAPK) for licorice zinc. Melanogenesis was induced by α-melanocyte-stimulating hormone *in vitro*. Cell counting kit-8, melanin content determination, and WB were performed to verify the inhibitory effect of licorice zinc on melanogenesis.

**Results:**

The present study showed that licorice zinc decreased melanin formation, cutaneous tissue injury, and the phosphorylation of JNK and P38MAPK, which was caused by UVB irradiation *in vivo*. *In vitro*, licorice zinc showed opposite effects from JNK/p38 activator. Meanwhile, tyrosinase-related protein-1, tyrosinase, and microphthalmia-associated transcription factor were decreased too.

**Conclusions:**

Licorice zinc induced a decrease in melanin synthesis by inhibiting the JNK and the P38MAPK signaling pathway, suggesting licorice zinc is a potential agent of anti-chloasma.

## Introduction

Chloasma is a common and acquired disorder of hyperpigmentation. It appears as light brown to dark and scattered among the sides of the cheekbones and the forehead^1^. The active melanocytes in the skin affected by hormones and ultraviolet (UV) irradiation cause chloasma formation. Chloasma formation implies epidermal and dermal melanin content increased^2^. Melanin is produced by melanocytes, which protect skin from UV irradiation. Melanin formation is a complex process involving tyrosinase, tyrosinase-related protein-1 (TRP-1), and microphthalmia-associated transcription factor (MITF)^3^^-^ 5. MITF is active by cAMP response element-binding protein (CREB), which is regulated by cAMP^3^^,^ 6. Moreover, it was recently reported that UV radiation and α-melanocyte-stimulating hormone (α-MSH) activate adenyl cyclase to increase cAMP^7^^-^ 9. It has been found that activating mitogen-activated protein kinases (p38 MAPK) and c-Jun-N-terminal (JNK) resulted in up-regulation of melanin content in B16F10 cells, and p38 MAPK was involved in MITF regulation^10^^,^ 11. So, JNK/p38 MAPK regulation is a viable target signaling pathway for melanogenesis.

The treatment of chloasma is still in great difficulty, due to the lack of effective and harmfulless therapeutic drugs. Tranexamic acid (TXA), an antifibrinolytic agent solvent which derived from lysine, prevents the lysis of fibrin clots by blocking the interaction between fibrinogen and fibrin^12^. Presently, TXA is the most widely used drug in clinical chloasma treatment due to outstanding therapeutic effect^13^. However, serious side effects limit its arbitrary application. These side effects include gastrointestinal symptoms, menstrual disorders, and venous thrombosis^14^. Thus, effective and harmfulless anti-chloasma agents are urgently needed.

Licorice (*Glycyrrhiza glabra* L.) is a perennial herb that belongs to the family Leguminosae^15^, and it has a prominent antioxidant capacity^16^. Licorice constituents present prominent tyrosinase inhibitory activity and anti-inflammatory activity too^17^^,^ 18. Inflammation is positively associated with melanogenesis^19^. These are important criteria to assess the inhibitory viability of melanogenesis. Furthermore, zinc is a trace element that plays a key role in more than 300 enzymes^20^, especially essential for cellular antioxidant defense^21^. Studies have shown that zinc has anti-inflammatory activity too^22^^,^ 23. Licorice zinc is a preparation of licorice combined with zinc, which plays the role of licorice and zinc. Briefly, licorice zinc has a whitening effect, which may have a prominent potential in the treatment of pigmented skin disease, especially chloasma. However, the mechanisms of licorice zinc are still poorly understood.

This study was set out to present new evidence that licorice zinc can be a clinical candidate drug for the treatment of chloasma, by investigating the effect of licorice zinc on melanogenesis and P38MAPK and JNK signaling pathway in the UVB irradiation-induced chloasma model of C57BL/6J mice and α-MSH-treated B16F10 cell.

## Methods

### Chloasma model manufacturing and drug treatment

A total of 39 female C57BL/6J mice (6-8 weeks) were obtained from the Jackson Laboratory. Chloasma modeling procedure as described as following. Mice were injected intramuscularly with progesterone (15 mg/kg) daily. Meanwhile, each mice had 3 × 3 cm back hair shaved, UVB irradiation (λ = 312 nm, 2 h/day) in C57BL/6J mice for 30 days. Then, mice were given 0.65 g/kg (UVB + L-licorice zinc) (n = 6), 1.3 g/kg (UVB + M-licorice zinc) (n = 6), 2.6 g/kg (UVB + H-licorice zinc) (n = 6) of licorice zinc (Best Pharmaceutical Co., LTD, China), and the positive drug TXA (Sigma, Japan) 250 mg, oral administration once a day, continuous processing for 14 days. The control group (control) (n = 6) and the model group (UVB) (n = 6) were given equal physiological saline. In addition, 26 g/kg licorice zinc administration (n = 3) for two days for preparation of licorice zinc positive serum. All animal experiments were conducted according to the ethical standards of experimental animals (Ethics Committee of Experimental Animals, Southwest Medical University, No. 20220112-001).

### Skin tissue HE and Masson-Fontana staining

All C57BL/6J mice were sacrificed. The mice skin of the shaved hair was taken, and stored in 4% formaldehyde solution for hematoxylin and eosin (HE) and Fontana-Masson staining. HE staining was carried out using an HE staining kit (Boster Biological Technology Company, China). Fontana-Masson staining was performed according to the product instructions (Junrui biotech Inc, China).

### Assessment of cell viability by CCK-8 assay

The assay cell counting kit-8 (CCK-8) (Beyotime, China) was used to measure whether licorice zinc positive serum (0, 5, 10, 20, and 40%) and 0.1 mM α-MSH (Bachem Bioscience Inc, United States of America) have toxic effects on the B16F10 cells. B16F10 cells (Procell, China) were cultured in Dulbecco’s Modified Eagle’s Medium (DMEM) (HyClone, China), and supplemented with 10% fetal bovine serum and 100 units/mL penicillin. Maintained 100 μg/mL streptomycin at 37 °C with a 5% CO_2_ atmosphere. B16F10 cells were cultured to the logarithmic growth phase. Then, 1×10^5^ cells/mL cell suspension was prepared with the pre-warmed medium and inoculated into a 96-well cell culture plate with a volume of 100 μL per/well. After the cells have adhered, the original medium was aspirated, divided cells into two groups, which added α-MSH or no added. Later, licorice zinc positive serum (0, 5, 10, 20, and 40%) was added in it, respectively. The next day, the CCK-8 solution was added 10 μL/well and incubated for another 4 h. It was measured at 450 nm.

### Melanin formation assay in vitro

1×10^5^ cells/mL B16F10 cells suspension was prepared with the pre-warmed medium and inoculated into a 96-well cell culture plate with a volume of 100 μL/well. Then, 0.1 mM α-MSH was added to every group, except the control one, and B16F10 cells were added to licorice zinc negative serum, the screened licorice zinc positive serum, and the screened licorice zinc positive serum + Anisomycin (Sigma, Japan), a JNK/p38 activator. The control group added phosphate-buffered saline (PBS). All groups were cultured for 24 h, the supernatant was discarded, and the groups were washed twice with PBS, received 100 μL 1 mol/L NaOH per well, 80 °C constant temperature water bath for 2 h. The optical density (OD) value of the sample was measured at 420 nm. The experiment was in quadruplicate.

### Western blot analysis

The skin of C57BL/6J mice and B16F10 cells were prepared for cellular lysates and performed Western blot. Proteins were separated by 10% sodium dodecyl sulfate-polyacrylamide gel electrophoresis (SDS-PAGE), and transferred electrophoretically onto polyvinyl difluoride (PVDF) membranes. The blots were blocked for 1 h at r.t with 4% bovine serum albumin (BSA). Then, probed with rabbit anti-mouse antibodies against JNK (1:1,000, abclonal, no. A5051, Wuhan, China), p-JNK (1:1,000, abclonal, no. Ap0276, Wuhan, China), P38MAPK (1:800, abclonal, no. A14401, Wuhan, China), p-P38MAPK (1:1,000, abclonal, no. Ap0526, Wuhan, China), TRP-1 (1:1,000, abclonal, no. A4016, Wuhan, China), tyrosinase (1:1,000, abclonal, no. A1254, Wuhan, China), MITF (1:1,000, abclonal, no. A11649, Wuhan, China), and β-actin (1:100,000, abclonal, no. AC026, Wuhan, China) at 4 °C overnight. After washing three times, the blots were subsequently incubated for 2 h at r.t with a goat horseradish peroxidase-conjugated secondary antibody. Then, washing four times for 10 min in Tris-buffered saline with Tween-20 (TBST), using a chemiluminescence detection system to detect the membranes. The intensity of the bands was quantified by ImageJ software.

### Statistical analysis

Results are expressed as the mean ± standard deviation (SD). Statistical comparisons between the control group and UVB/α-MSH model groups by Student’s t-test, and comparisons in multiple groups were carried out with one-way analysis of variance (ANOVA) by Tukey’s post-hoc tests. Statistical analyses were performed using GraphPad Prism 9.1.2 software. Statistical significance was defined at p < 0.05. All the experiments were performed at least three times.

## Results

### Licorice zinc reduces skin tissue damage

**Figure 1:**
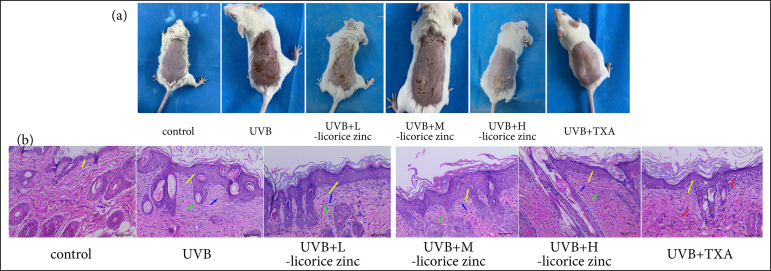
Licorice zinc inhibited UVB-induced necrosis of collagen fibers in dermis. C57BL/6J mice skin was treated with UVB, mice were given licorice zinc (0.65 g/kg, 1.3 g/kg, 2.6 g/kg) and 250mg TXA once a day.

UVB radiation caused cutaneous tissue injury, scab format and skin got blacker compared to the control group. After licorice zinc and TXA were treated, cutaneous tissue injury got better. Among them, 1.3, and 2.6 g/kg licorice zinc, and 250 mg TXA had a good therapeutic effect; the skin of C57BL/6J mice became smooth and white (Fig. 1a). However, the cuticle of the 1.3, and 2.6 g/kg licorice zinc treated group was thicker than TXA treated. HE results (Fig. 1b) showed that UVB irradiation induced a severe epidermal hyperplasia, hair follicles necrosis, and fibrous tissue hyperplasia. In an attempt to identify an appropriate drug for single-dose, we examined 0.65, 1.3, and 2.6 g/kg licorice zinc, respectively. Epidermal and fibrous hyperplasia decreased with increasing drug dose compared to the UVB treated group. Interestingly, 250-mg TXA has no better therapeutic effect compared to 6 g licorice zinc.

Each group of skin tissue was stained for HE staining, under an IX70 microscope. Bars=50μm.

### Licorice zinc inhibits melanophore formation

**Figure 2:**
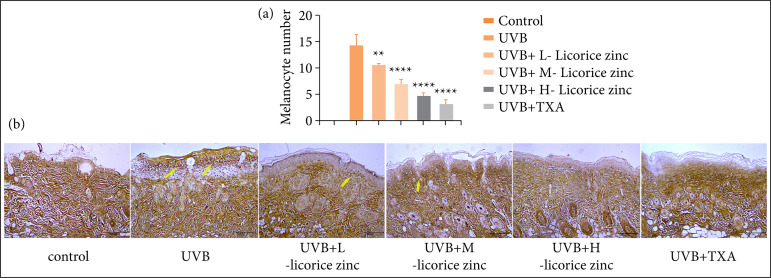
Licorice zinc inhibited UVB-induced melanocyte formation C57BL/6J mice skin was treated with UVB, mice were given licorice zinc (0.65 g/kg, 1.3 g/kg, 2.6 g/kg) and 250mg tranexamic acid once a day. **(a)** The melanocyte number was counted per field of microscope and the number of the control group was zero. **(b)** Each group of skin tissue was stained for Masson-Fontana staining, under an IX70 microscope.

Fontana-Masson staining was performed to verify whether licorice zinc could inhibit melanophore formation. The results shown in Fig. 2 revealed no melanophore and a regular skin structure in the control group, while the UVB treated group showed melanophores enriched in the epidermis, and the UVB + TXA treated group showed little melanophores deposition. Especially, melanophores were significantly reduced as the licorice zinc treatment concentration increased (Figs. 2a and 2b). The statistical results of melanophores were shown in Fig. 2a. Results indicated UVB + L-licorice zinc treated group reduced the melanophores number (p < 0.01), and the UVB + M-licorice zinc treated group, UVB + H-licorice zinc treated group and UVB + TXA treated group all have significant differences for melanophores number reduced (p < 0.0001). All were compared to UVB treated group.

Bars represent the mean ± S.D. from three independent experiments. **p<0.01 and ****p<0.0001 compared with UVB-treated group. Bars=100μm

### Licorice zinc inhibits the activation of P38MAPK and JNK pathway in vivo

**Figure 3:**
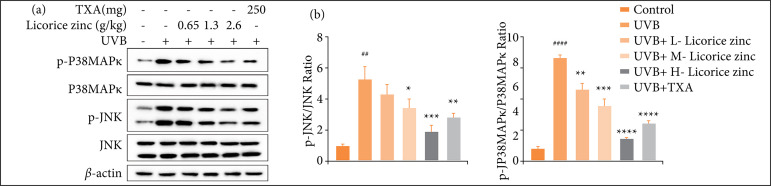
Licorice zinc inhibited UVB-induced JNK/P38MAPK signaling pathway activation. C57BL/6J mice skin was treated with UVB, mice were given licorice zinc (0.65 g/kg, 1.3 g/kg, 2.6 g/kg) and 250mg tranexamic acid once a day. **(a)** JNK/ P38MAPK signaling pathway-related proteins were analyzed by western blotting using antibodies against JNK, p-JNK, P38MAPK, and p-P38MAPK, β-actin served as a loading control. **(b)**The ratio of p-JNK/ JNK and p-P38MAPK/ P38MAPK were quantified by western blotting analysis and normalized to control.

The expressions of P38MAPK and JNK signaling pathway-related proteins including JNK, p-JNK, P38MAPK, and p-P38MAPK were analyzed quantitatively using WB (Fig. 3a). The data indicated that treatment groups inhibited the expression of p-JNK, and p-P38MAPK, and the inhibitory effects were also in a dose-dependent manner. Notably, the ratio of p-JNK /JNK and p-P38MAPK/ P38MAPK was quantified; they both were increased significantly after UVB irradiation, and were decreased after being given different concentrations of licorice zinc and TXA (Fig. 3b). Particularly, 2.6-g licorice zinc exhibited the best inhibition of these proteins.

Bars represent the mean ± S.D. from three independent experiments. ####p<0.0001 compared with control group, *p<0.05, **p<0.01, ***p<0.001, and ****p<0.0001 compared wi th UVB-treated group.

### Licorice zinc positive serum screening

**Figure 4:**
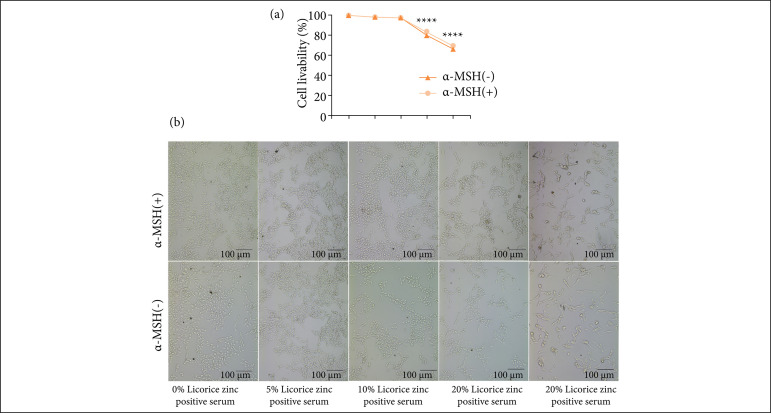
Screened of positive serum concentration of licorice zinc for B16F10 in vitro test. B16F10 cells were cultured for 24 h, then treated with 0%, 5%, 10%, 20%, 40% licorice zinc positive serum for 72 h with or without 0.1 mM α-MSH. **(a)** Cell viability was measured by CCK-8 assay, **(b)** B16F10 cells proliferation under IX70 microscope.

To eliminate the inhibitory effects of licorice zinc positive serum and α-MSH on B16F10 cell, CCK-8 assay was performed. The data showed that the cell viability of B16F10 cells was not distinctly reduced by α-MSH with concentrations of 0.1 μM, indicating that 0.1 μM α-MSH was nontoxic to B16F10 cells (Fig. 4a). Additionally, the data and figure showed that 10% licorice zinc positive serum was the maximum concentration with no effect on B16F10 cells activity (Figs. 4a and 4b), which was appropriate for subsequent cells test.

****p<0.0001 compared with 0% licorice zinc positive serum. Bars=100μm.

### Inhibition of P38MAPK and JNK pathway restrained melanin formation in vitro

**Figure 5:**
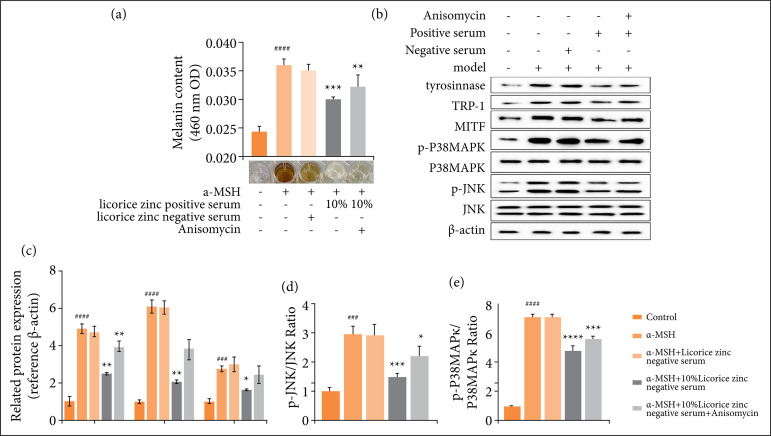
Inhibition JNK pathway restrained UVB--induced melanin formation. After B16F10 cells were cultured for 24 h, cells were treated with 0.1 mM α-MSH for 72 h, meanwhile, cells were treated with licorice zinc negative serum, 10% licorice zinc positive serum, and 10% licorice zinc positive serum+Anisomycin. The Control group was cultured with nothing except medium all along. **(a)** The content of melanin was measured by sodium hydroxide dissolution. **(b)** JNK/ P38MAPK signaling pathway and melanin productionrelated protein were an alyzed by western blotting using antibodies against JNK, p-JNK, P38MAPK, pP38MAPK, TRP-1, tyrosinase, and MTIF. β-actin served as a loading control. **(c)** The relative density of TRP1, tyrosinase, and MTIF, and the ratio of p-JNK/ JNK and p-P38MAPK/ P38MAPK were quantified by western blotting analysis and normalized to control.

To elucidate the effects of inhibition of P38MAPK and JNK pathway on α-MSH-induced melanin formation in B16F10 cells, 10% licorice zinc positive serum combined with or not with Anisomycin was used to treat α-MSH-induced B16F10 cells. The content of melanin was measured by sodium hydroxide dissolution (Fig. 5a), and Western blots were used to examine the expression levels of melanogenesis-related proteins (Figs. 5b, 5c, and 5d). As Fig. 5a has shown, 10% licorice zinc positive serum significantly reduced the melanin content in the B16F10 cells (Fig. 5a). Compared to α-MSH treated group, the expression levels of all the proteins which involved in the melanin biosynthetic pathway were significantly reduced, involved TRP-1, tyrosinase and MITF (Fig. 5c); and the ratio of p-JNK/ JNK and p-P38MAPK/ P38MAPK was down-regulated significantly (Fig. 5d). In addition, Anisomycin reversed the situation of 10% licorice zinc positive serum treatment in α-MSH treatment group, that the melanin content, the expression levels of melanin biosynthetic related protein, and the ratio of p-JNK/ JNK and p-P38MAPK/ P38MAPK were up-regulated all.

Bars represent the mean ± S.D. from three independent experiments. ####p<0.0001 compared with control group, *p<0.05, **p<0.01, ***p<0.001, and ****p<0.0001 compared with α-MSH -treated group.

## Discussion

UV radiation is one of the major extrinsic factors inducing hyperpigmentation skin disorder. The UV radiation has three wavelength ranges: UVA, UVB, and UVC. Particularly UVB (290-320 nm) is considered the root cause of skin photoaging^24^. Excessive UVB radiation causes inflammatory responses, cutaneous tissue injury, cellular oxidation, and cutaneous melanogenesis^25^. Thus, we modeled chloasma by UVB radiation and progesterone injection. In this study, we are the first to investigate the mechanisms of inhibiting chloasma formation for licorice zinc. Data showed that licorice zinc treatment could reduce the melanin content and cutaneous tissue injury. This suggests that licorice zinc may be a potential drug for the treatment of chloasma. Compared with licorice zinc treatment, TXA treated group presented necroptosis of collagen fibers and epidermis, inferring that licorice zinc has a safer effect on chloasma treatment.

There is a growing body of evidence indicating that the P38MAPK and JNK signaling pathway contributes to melanogenesis via colaborating to inflammatory and oxidants^26^^,^ 27. Reactive oxygen species (ROS) production and inflammatory mediators were suppressed when P38MAPK and JNK signaling pathway was suppressed, what is more, the current study indicated that inhibition of inflammatory response and oxidative stress could reduce melanogenesis^19^. Therefore, the inhibition of the P38MAPK and JNK signaling pathway could inhibit melanogenesis. This study has found that licorice zinc could inhibit the activating of P38MAPK and JNK signaling pathways and reduce melanin formation in C57BL/6J mice, which corresponds to previous reports.

Furthermore, to determine the mechanism of the way licorice zinc works, B16F10 cells experiment was performed. α-MSH is the main intrinsic stimulator of melanogenesis, which is commonly used in anti-melanogenesis studies^28^. In the course of the screening process for optimal concentration of licorice zinc serum, we found that the ratio of 10% was the most suitable addition amount for B16F10 cells treated. We determined that 10% licorice zinc serum and 0.1 mM α-MSH exhibited no cytotoxicity in B16F10 cells. Thus, the concentration was used for subsequent experiments. In this study, α-MSH-induced a melanin synthesis in B16F10 cells and an up-regulation of the ratio of p-JNK/JNK and p-P38MAPK/P38MAPK, indicating that the activation of P38MAPK and JNK pathway contribute to melanin synthesis. However, the situation was reversed by licorice zinc, while Anisomycin weakened the effects of licorice zinc. We inferred that licorice zinc may target the P38MAPK and JNK signaling pathway to inhibit melanin synthesis.

Reported enhanced activation of P38MAPK and JNK caused tyrosinase transcription increase^29^. On one hand, P38MAPK activation would up-regulate MITF^30^. On the other hand, MITF is crucial for TRP-1 and tyrosinase, which connect to melanocyte survival^29^. Therefore, inhibition of P38MAPK and the JNK signaling pathway may inhibit melanogenesis. It may be a hopeful therapeutic target for the treatment of chloasma. In this study, α-MSH significantly activated the P38MAPK and JNK signaling pathways and increased the expression of TRP-1, tyrosinase, and MITF, causing melanin formation. After treatment, our results showed that licorice zinc significantly inhibited JNK and P38MAPK phosphorylation. TRP-1, tyrosinase, and MITF are enzymes known as markers of melanogenesis, licorice zinc down-regulated it all. Conversely, Anisomycin, a specific JNK activator, showed a reverse pattern. So that we attributed licorice zinc may target JNK or its upstream to inhibit melanogenesis. Furthermore, TRP-1, tyrosinase, and MITF were also regulated by PI3K/AKT pathway^31^. Therefore, melanogenesis regulation is a complex variety of regulation networks rather than a single regulator, while the detailed mechanism of this is still unclear.

Encouragingly, the inhibitory effect of licorice zinc is similar to that of TXA. Considering that the P38MAPK and JNK signaling pathway is the key pathway of melanogenesis, we conclude that licorice zinc down-regulating TRP-1, tyrosinase, and MITF via inhibiting P38MAPK and JNK phosphorylation, ultimately, caused melanin to reduce.

## Conclusions

In conclusion, these findings illustrated that licorice zinc inhibited UVB-induced and α-MSH-induced melanogenesis by suppressing P38MAPK and the JNK signaling pathway. The study suggested that licorice zinc could be used as a pigmented disease therapeutic drug since a low concentration of licorice zinc could inhibit melanogenesis.
