# ERRATA

**DOI:** 10.1590/acb371002.errata

**Published:** 2023-05-01

In the manuscript Licorice zinc suppresses melanogenesis via inhibiting the activation of P38MAPK and JNK signaling pathway in C57BL/6J mice skin, which DOI is: https://doi.org/10.1590/acb371002, published in the journal **Acta Cirúrgica Brasileira**, 2022;37(10)e371002:

On the title in the main page and on the header in every even page;

**Page 1**

On the third line of the Abstract;

**Page 2**

In the last line of the 3rd paragraph;

In the 1st and 3rd line of the 1st paragraph of Methods;

In the 1st line of the 2nd paragraph in Methods;

**Page 3**

In the first line of the Subtitle: Western blot analysis;

In the 3rd line of the first paragraph of Results;

**Page 4**

In the Caption of the Figure 1 and 2;

**Page 5**

In the Caption of the Figure 3;

**Page 7**

In the last line of the 2nd paragraph

Instead of

C57BL/6J

Should be

Balb/c

